# Antarctic Krill Oil Attenuates Oxidative Stress via the KEAP1-NRF2 Signaling in Patients with Coronary Heart Disease

**DOI:** 10.1155/2020/9534137

**Published:** 2020-10-07

**Authors:** Chengfei Wen, Mi Jiang, Weixin Huang, Shumei Liu

**Affiliations:** ^1^Department of Cardiology, The First Hospital of Jilin University, Changchun 130021, China; ^2^Department of Cardiac Surgery, The First Hospital of Jilin University, Changchun 130021, China; ^3^Department of Geriatrics, The First Hospital of Jilin University, Changchun 130021, China; ^4^Department of Endocrinology, The First Hospital of Jilin University, Changchun 130031, China

## Abstract

**Background:**

Antarctic krill oil (AKO) has strong antioxidant activities and is effective for alleviating coronary heart disease (CHD). Kelch-like ECH-associated protein 1-NF-E2-related factor 2 (KEAP1-NRF2) axis is a crucial antioxidant signaling pathway. Thus, AKO may exert its antioxidant effects on CHD patients via KEAP1-NRF2 signaling.

**Methods:**

AKO fatty acid (FA) profiles were analyzed by using gas chromatography (GC). One hundred CHD patients were divided into the intervention (IG, AKO) and control (CG, placebo) groups. Before and after 1, 2, and 3 months of intervention, we measured serum levels of reactive oxygen species (ROS), 8-hydroxy-2-deoxyguanosine (8-OHdG), nitric oxide (NO), malondialdehyde (MDA), superoxide dismutase (SOD), reduced glutathione (GSH), and glutathione peroxidase (GPx), and KEAP1 and NRF2 levels in peripheral blood leukocytes (PBLs). Serum FAs were measured by GC at baseline and after 3-month intervention.

**Results:**

AKO contains rich eicosapentaenoic acid (EPA) and docosahexaenoic acid (DHA), which is more than 27% of total FA. The levels of EPA and DHA, KEAP1, and NRF2 in the IG group were higher than those in the CG group (*p* < 0.05). Serum levels of ROS, 8-OHdG, NO, and MDA in the IG group were lower than those in the CG group, whereas the levels of SOD, GSH, and GPx in the IG group were higher than those in the CG group (*p* < 0.05). Serum levels of saturated fatty acids (UFA) in the IG group were higher than those in the CG group, whereas reverse results were obtained for the levels of saturated fatty acids (SFA). Serum levels of EPA and DHA had a strong negative relationship with the level of ROS, whereas the ROS level had a strong negative relationship with the levels of KEAP1-NRF2.

**Conclusion:**

AKO increases antioxidant capacities of CHD patients via the KEAP1-NRF2 signaling in the PBL.

## 1. Introduction

Coronary heart disease (CHD) is the leading cause of global mortality [1]. Treating CHD is challenging mainly due to poor prognosis and clinical outcomes affected by systemic hypertension [2], atherosclerosis [3], and inflammatory factors [4]. Oxidative stress often induces hypertension [5], atherosclerosis [6], and inflammatory responses [7]. A high level of oxidative stress is closely associated with CHD progression [8], and antioxidant therapy has become a potential therapeutic option in the prevention of CHD risk [9, 10].

Previous work reported the protective role of krill oil intake against the risk of cardiovascular disease [11]. However, the clinical effects of AKO on CHD and related molecular mechanism remained widely unknown. EPA and DHA were reported to reduce the risks of heart disease for decades, and there has been increasing evidence that EPA and DHA consumption results in different clinical and molecular effects [12]. Antarctic krill oil (AKO) is rich with long-chain polyunsaturated fatty acids (LCPUFAs), such as eicosapentaenoic acid (EPA) and docosahexaenoic acid (DHA) [13], which may be especially important in antioxidant defenses. EPA showed strong antioxidant activity with obvious inhibitory function for alpha-diphenyl-beta-picrylhydrazyl (DPPH) radical and 2, 20-azinobis-3-ethylbenzothiazoline-6-sulfonic acid (ABTS) radical cation [14]. DHA improved antioxidant status and reduced deoxyribonucleic acid damage [15]. A randomized and double-blind parallel arm trial in overweight and obese patients showed that 4 weeks of AKO supplementation increased plasma contents of EPA and DHA without adverse effects on safety-related performance [16]. The results suggest that AKO may have protective functions for CHD patients by increasing antioxidant properties via EPA and DHA.

Kelch-like ECH-associated protein 1-NF-E2-related factor 2 (KEAP1-NRF2) is a widely reported antioxidant signaling pathway [17–20]. AKO may also exert its antioxidant activity by affecting KEAP1-NRF2 signaling via EPA and DHA. Thus, this study aimed to measure the EPA and DHA contents in AKO and explore the antioxidant effects of AKO dietary intervention on CHD patients by measuring the levels of oxidative stress-related parameters and KEAP1-NRF2.

## 2. Materials and Methods

### 2.1. Measurement of Total Fatty Acid (FA) Composition in AKO

AKO was purchased from Hailisheng Group Co., Ltd. (Zhoushan, Zhejiang, China) and stored at 20°C. The FA composition in AKO was analyzed using an Agilent 7820 gas chromatograph (Agilent; Wilmington, DE, USA) with the following method: 20 different patches of samples were tested in spiral test tubes, and 1 mL of a sodium hydroxide-methanol solution (2 mol/L) was pipetted into each tube and methylated in a water bath at 80°C for 45 min. After the reaction solution was cooled on ice, 2 mL of sulfuric acid-methanol solution (0.5 mol/L) was added, and the methylated reaction was further performed in a water bath at 80°C for 45 min; after taking it out again for cooling, 1 mL of chromatographic pure hexane was added for fatty acid methyl ester (FAME) extraction, and then saturated sodium chloride solution was added to promote the separation of the solution system. A small amount of anhydrous sodium sulfate was added to one half mL of centrifuge tube to dehydrate it. After passing through the membrane, the organic phase was transferred into a sample bottle for gas chromatography analysis. Gas chromatography conditions were set as follows: chromatographic column, trace TR-FAME capillary column (0.2 *μ*m, 60 m × 0.25 mm); inlet temperature, 230°C; detector temperature, 250°C; carrier gas flow rate, 1 mL/min; split ratio, 1 : 100. The programmed heating conditions were set as follows: the initial column temperature was maintained at 120°C for 2 min; the temperature was increased at a rate of 5°C/min to 175°C, and the temperature was increased at a rate of 10°C/min to 220°C and finally hold for 10 min. 37-component FA methyl ester standard mixture (cat. CDAA-252795-MIX-1 mL) was purchased from ANPEL Scientific Instrument Co. Ltd. (Shanghai, China). Under the above chromatographic condition, a standard mixture of FAME was injected into the gas chromatograph, the chromatographic peaks were qualitatively determined, and the area normalization was used to determine the content of individual FA in the total FA mass (%, wt). All FAMEs from AKO were normalized according to the FAME standards. The content of FA was calculated as 100% (w/w) = content of each FA × 100/total relative FA contents for all FAs [21].

### 2.2. Participants

Before the experiments, all processes were approved by the Ethic Committee of The First Hospital of Jilin University (approval no. FHJLA20XY569) and consistent with the World Medical Association Declaration of Helsinki. From December 2017 to March 2018, 328 CHD patients visited our hospital and were recruited in our hospital. The written consent form was obtained from each subject.

#### 2.2.1. Inclusion Criteria

CHD patients were proven by clinic, electrocardiogram, and selective coronary arteriography according to the diagnostic criteria for CHD [22, 23] and the standard questionnaire [24], and the patients aged more than 18 years.

#### 2.2.2. Exclusion Criteria

The patients had mental diseases and were difficult to be communicated. The patients dropped out in time and would replenish the sample size in time. The patients had other serious diseases, such as cancers or liver diseases. Some patients took antioxidant drugs and healthy products with antioxidant activities.

#### 2.2.3. Randomization

After inclusion and exclusion criteria, 100 CHD patients were randomly assigned into the IG (received 2 g of AKO daily) and the CG (placebo) groups by using the random number generated by a computer, and the allocation ratio was 1 : 1. The dietary intervention periods were 3 months.

### 2.3. Measurement of Serum FA

All participants were fasted for 9 h, and 5 mL of peripheral venous blood was collected into the BD Vacutainer tube. Blood samples were centrifuged at 5000  × g for 10 min, and serum was obtained. Lipids were extracted along with triheptadecanoyl-glycerol, and FAMEs were made by using 6% H_2_SO_4_ in methanol [25]. Individual FAs were separated and analyzed by using the above chromatographic condition. The contents of saturated fatty acids (SFA), monounsaturated fatty acids (MUFA), and polyunsaturated fatty acids (PUFA) were compared among the IG and CG groups.

### 2.4. Measurement of Serum Oxidative Parameters

Serum reactive oxygen species (ROS) was measured using N,N-diethyl-para-phenylenediamine (DEPPD) (Sigma-Aldrich, USA) [26]. Serum 8-hydroxy-2-deoxyguanosine (8-OHdG) was measured using ELISA kit from the Japan Institute for the Control of Aging (Fukuroi, Shizuoka). Solution I (4.37 *μ*M of ferrous sulfate in 0.1 M sodium acetate buffer, pH 4.8) and solution II (100 *μ*g/ml of DEPPD in 0.1 M sodium acetate buffer, pH 4.8) were used to construct the calibration curve of ROS. Five *μ*l of H_2_O_2_ was added to 140 *μ*L of 0.1 M sodium acetate buffer (pH 4.8) in each well of a 96-well microtiter plate and incubated at 37°C after 5 min. One hundred *μ*L of solution I and 5 *μ*L of solution II were mixed, added to each cell and incubated at 37°C for 5 min. The absorbing values were measured at 505 nm by using a spectrophotometric plate at 15 s intervals for 3 min. A calibration curve was plotted according to the value variation (min) corresponding to the concentration of H_2_O_2_ (1 unit = 1.0 mg H_2_O_2_/L). Five *μ*L of serum was added to 140 *μ*L of 0.1 M sodium acetate buffer (pH 4.8) in each well of a 96-well microtiter plate and incubated at 37°C after 5 min. One hundred *μ*L of solution I and 5 *μ*L of solution II were mixed and added to the cuvette. The cuvette was incubated for 5 min at 37°C. The absorbing values were measured at 505 nm for 3 min. The ROS levels were calculated according to the calibration curve.

A series of 8-OHdG standards were diluted in the concentration ranging from 0 ng/mL to 20 ng/mL by using the assay diluent. The serum sample was diluted in assay diluent and filtered through a 0.45 *μ*m filter. Fifty *μ*L of the sample or 8-OHdG standard was added to the wells of the 8-OHdG conjugate coated plate and incubated for 10 min at room temperature on an orbital shaker. Fifty *μ*L of the diluted anti-8-OHdG antibody was added to each well and incubated at room temperature for 1 h on an orbital shaker. The wells were washed with 250 *μ*L of washing buffer for 3 times. One hundred *μ*L of the diluted secondary antibody-enzyme conjugate was added to each well and incubated at room temperature for 1 hour on an orbital shaker. The wells were washed for 3 times according the above method. One hundred *μ*L of substrate solution was added to each well and incubated at room temperature on an orbital shaker for 20–30 min according the color changes. One hundred *μ*L of stop solution was added to each well, and absorbing values were measured at 450 nm. A standard curve was constructed by using the series of 8-OHdG standards, and the contents of 8-OHdG were calculated based on the standard curve.

Serum nitric oxide (NO) was determined using the Griess method (Invitrogen, CA, USA). Serum malondialdehyde (MDA) was measured using the thiobarbituric acid-trichloroacetic acid (TBA-TCA) method [27]. Serum superoxide dismutase (SOD) activity was analyzed by using a SOD kit (Nanjing Jiancheng Bioengineering Institute). Serum reduced glutathione (GSH) was determined using ELISA reagent kit from Shanghai BlueGene Biotech Co. (Shanghai, China). Serum glutathione peroxidase (GPx) activity was measured using the Cayman GPx Assay Kit (Cayman Chemical Company, Ann Arbor, MI, USA). Serum SOD was analyzed by examining the inhibition of hydroxylamine oxidation using a SOD-detection assay kit (Jiancheng Bioengineering, Nanjing, China) [28]. Hydroxylamine oxidation capacity was measured at 450 by using a spectrophotometer (Shimadzu, Japan). One unit of SOD was defined as the amount of enzyme that inhibited hydroxylamine oxidation by 50%. Serum GSH was determined by using a kit from Shanghai BlueGene Biotech Co. (Shanghai, China) by using the spectrophotometric method [29]. The levels of yellow products were measured at 405 nm using a spectrophotometer (Shimadzu, Japan). Serum glutathione peroxidase (GPx) was measured by detecting the changes of NADPH to NADP^+^ content using a Cayman GPx Assay Kit (Cayman Chemical Company, Ann Arbor, MI, USA) [30]. NADPH concentration was measured at 340 nm by using a spectrophotometer (Shimadzu, Japan). One unit of GPx was defined as *μ*m of NADPH oxidized per min.

### 2.5. Quantitative Real-Time PCR (qRT-PCR) Analysis

Four mL of heparinized blood was obtained from each subject, and peripheral blood leukocytes (PBLs) were isolated by using density-gradient centrifugation over Histopaque-1077 (Sigma-Aldrich, USA). Total RNA was isolated by using the RNA extraction kit (Sanggon Biotech Co., Ltd. Shanghai, China). One *μ*g of RNA was reversely transcribed by using Transcriptor First Strand cDNA Synthesis Kit (Roche, Germany). The following primers were synthesized by TaKaRa (Dalian, China): KEAP1 (forward primer, 5′-catggcaaccgcaccttcag-3′ and reverse primer, 5′-ctcagtggaggcgtacatca-3′), NRF2 (forward primer, 5′-aacacacggtccacagctc-3′and reverse primer, 5′-tcttgcctccaaagtatgtcaa-3′), and *β*-actin (forward primer, 5′-tcctccctggagaagagcta-3′ and reverse primer, 5′-gcactgtgttggcatacagg-3′) as an internal control. The qRT-PCR was performed as follows: 1 cycle of 94°C for 50 s, followed by 40 cycles of 94°C for 15 s, 60°C for 15 s, and 1 cycle of 65°C for 50 s. Relative mRNA levels of NRF2 and KEAP1 were normalized to the level of *β*-actin and analyzed using the 2^−ΔΔC T^ method. Fold change in relative mRNA levels was analyzed based on the 2^−∆∆Ct^ method, where ∆∆Ct = 2^−ΔΔCt^ = [(∆Ct target gene − ∆Ct control) in the IG group − [(∆Ct target gene − ∆Ct control) in the CG group].

### 2.6. Western Blot

PBLs were lysed with the buffer containing 50 mM EDTA, 50 mM NaCl, 1% SDS, and 200 *μ*g/ml proteinase K and treated at 37°C for 6 h [31]. The proteins were separated by 10% polyacrylamide gel and transferred to polyvinylidenedifluoride (PVDF) membranes (Millipore, CA, USA). The membrane was blocked with 5% nonfat milk and incubated with NRF2 (ab31163, 1 : 2000, Abcam), KEAP1 (ab139729, 1 : 1000, Abcam), and *β*-actin antibodies (ab8227, 1 : 5000, Abcam) from Abcam overnight at 4°C. The membrane was then incubated with secondary antibody goat anti-rabbit IgG H&L (HRP) (ab6721, 1 : 3000) for 2 h at room temperature. Immunoreactivity bands were visualized by using ECL Prime (GE Healthcare Science) and analyzed using a VersaDoc 5000 System (Bio-Rad). Quantification was estimated using ImageJ software (version 1.42; the National Institutes of Health, Bethesda, MD, USA).

### 2.7. Immunofluorescence Staining

The nuclear translocation of NRF2 and KEAP1 was detected via immunofluorescence staining. PBL cells (1 × 10^4^/ml) were cultured in laser confocal Petri dishes, treated with andrographolide sodium bisulfate (ASB) for 1 d, and irradiated with UV (300 *μ*W/cm2 sec x 300 sec). PBL were washed 4 times with PBS and fixed with 4% paraformaldehyde for 20 min at room temperature, permeabilized with 1% Triton *X* for 20 min, and then washed and blocked with 5% goat serum (Invitrogen, Frederick, MD, USA) for half an hour. PBL were incubated with primary antibodies (NRF2, 1 : 2000, and KEAP1, 1 : 2000) at 4°C overnight, washed with TBST from G-Biosciences (St. Louis, MO, USA), and incubated with a secondary antibody conjugated to a fluorochrome (FITC, Enzo Life Sciences, NY, USA) for 2 h at room temperature. PBL were stained with DAPI (10 *μ*g/ml) for 10 min, washed with PBST 3 times, and drawn on the slides with a drop of the fluorescent mounting medium. The antibody localization was visualized using a fluorescence microscope (EVOS; Life Technologies, USA).

### 2.8. Statistical Analysis

All data were analyzed using SPSS 22 statistical software (IBM SPSS, Armonk, NY, USA). Two groups of patients in the general social demographic data were expressed in the qualitative data using the f-test. The analysis of variance (ANOVA) was performed by using the Kruskal–Wallis rank sum test. The two-sided test was used to detect overall differences between the two groups, and the test level *a* = 0.05. The analysis of one-way ANOVA was performed to test multiple comparisons between the two groups. Pearson's correlation coefficient test was used to explore the statistical relationship between serum levels of EPA or DHA and ROS level, and between ROS level and KEAP1, or NRF2 level in PBL.

## 3. Results

### 3.1. The FA Components of AKO

**Figure 1:**
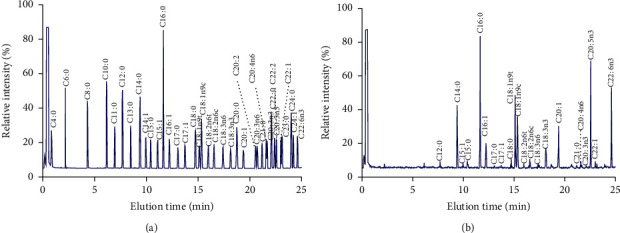
Gas chromatography analysis of fatty acid methyl esters: (a) fatty acid methyl ester standard mixture (37 components, C4–C24); (b) fatty acid methyl esters from AKO.

According to FAME standard (Figure 1(a)), krill oil boasts an impressive FA profile and contains two omega-3 PUFA, eicosapentaenoic acid (EPA) and docosahexaenoic acid (DHA), which were more than 27% of total FAs (Figure 1(b) and Table 1). As a plankton, the lipids in Antarctic krill mainly come from the food marine single-cell algae, such as diatoms with high levels of EPA and flagellates with high levels of DHA [32–35], which is why Antarctic krill oil contains high levels of EPA and DHA.

### 3.2. Demographic Data

In this study, 100 CHD patients who met the selection criteria were included. In the IG group, there were 26 males and 24 females, aged 52–69 years, and average was 60.30 ± 8.40 years old. In the CG group, there were 24 males and 26 females, aged 47–71 years, and average was 58.56 ± 12.23 years. Demographic data included age, gender, smoking history, preoperative nutritional status, and BMI (the body mass index). There was no statistically significant difference during surgery (Table 2, *p* > 0.05).

### 3.3. AKO Improved Antioxidant Properties in CHD Patients

**Figure 2:**
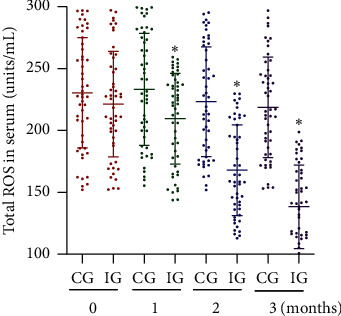
Serum level of reactive oxygen species (ROS). IG, Antarctic krill oil intervention group; CG, common-care group. *n* = 50 for each group, and intervention duration was 3 months. The statistical difference was significant if *p* < 0.05.

Before AKO intervention, the statistical difference for ROS levels was insignificant between the two groups (Figure 2, *p* > 0.05). After AKO intervention, ROS level reduced in the IG group with the time going, whereas the level changed little in the CG group. ROS level in the IG group was lower than that in the CG group (Figure 2, *p* < 0.05). The results suggest that AKO intervention reduces oxidative stress in the CHD patients.

**Figure 3:**
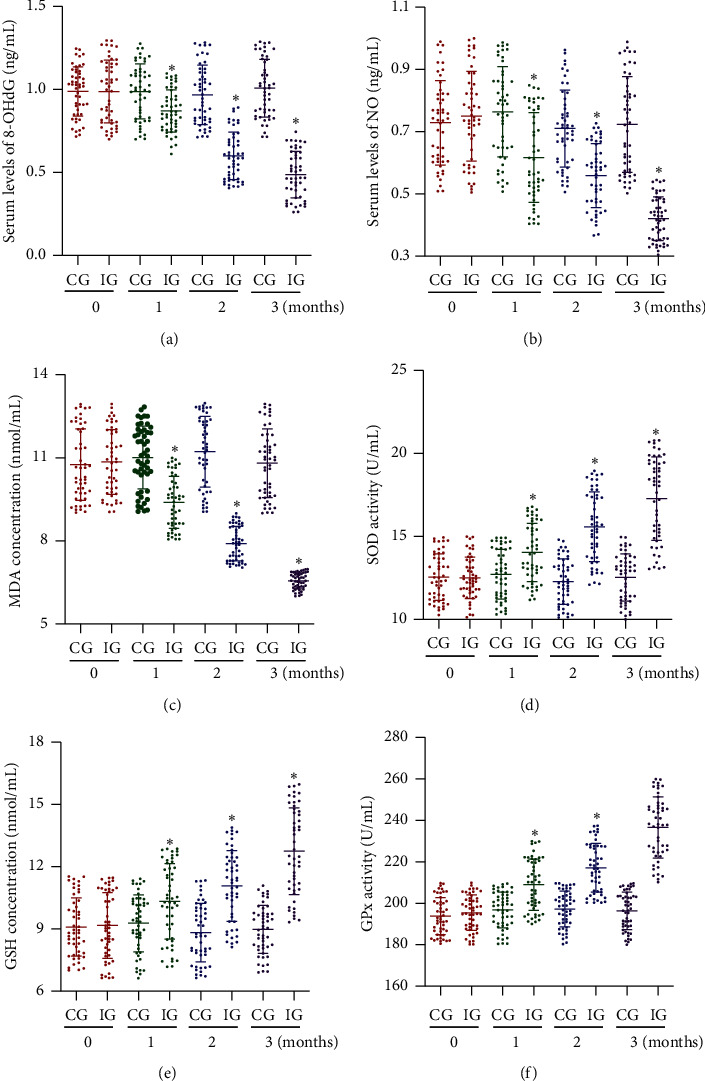
Serum levels of oxidative stress biomarkers: (a) 8-OHdG, 8-hydroxy-2-deoxyguanosine; (b) NO, nitric oxide; (c) MDA, malondialdehyde; (d) SOD, superoxide dismutase; (e) GSH, reduced glutathione; (f) GPx, glutathione peroxidase. IG, Antarctic krill oil intervention group; CG, common-care group. *n* = 50 for each group, and intervention duration was 3 months. The statistical difference was significant if *p* < 0.05.

In the similar cases, before AKO intervention, the statistical differences for the serum levels of 8-OHdG (Figure 3(a))), NO (Figure 3(b)), and MDA (Figure 3(c)) were insignificant between the two groups (*p* > 0.05). After AKO intervention, the levels were reduced in the IG group with the time going, whereas the levels were changed little in the CG group. The levels of 8-OHdG (Figure 3(a)), NO (Figure 3(b)), and MDA (Figure 3(c)) in the IG group were lower than those in the CG group (*p* < 0.05). The results also suggest that AKO intervention reduces oxidative stress in the CHD patients.

For the antioxidant biomarkers, the statistical differences for the serum levels of SOD (Figure 3(d)), GSH (Figure 3(e)), and GPx (Figure 3(f)) were insignificant between the two groups (*p* > 0.05). In contrast, after AKO intervention, the levels were increased in the IG group with the time going, whereas the levels changed little in the CG group. The serum levels of SOD (Figure 3(d)), GSH (Figure 3(e)), and GPx (Figure 3(f)) in the IG group were higher than those in the CG group (*p* < 0.05). The results also suggest that AKO intervention increases antioxidant capacities in the CHD patients.

### 3.4. AKO Improved Serum Levels of EPA and DHA in CHD Patients

**Figure 4:**
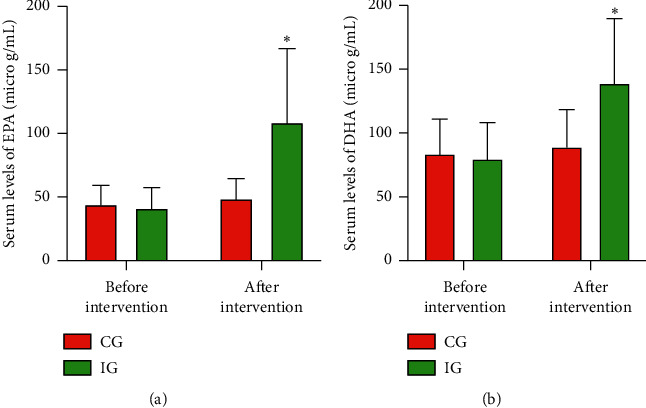
Serum levels of EPA and DHA in the CHD patients: (a) EPA; (b) DHA. IG, Antarctic krill oil intervention group; CG, common-care group. *n* = 10 in each group, and intervention duration was 3 months. The statistical difference was significant if *p* < 0.05.

Before AKO intervention, the statistical differences for the serum levels of EPA (Figure 4(a)) and DHA (Figure 4(b)) were insignificant between the two groups (*p* > 0.05). After AKO intervention, serum levels of EPA (Figure 4(a)) and DHA (Figure 4(b)) were significantly increased in the IG group, whereas the level changed little in the CG group. The serum levels of EPA and DHA in the IG group were significantly higher than that in the CG group (*p* < 0.05). The results suggest that AKO intervention increased the serum level of EPA and DHA in the CHD patients.

### 3.5. AKO Increased Relative mRNA Levels of KEAP1 and NRF2 in CHD Patients

**Figure 5:**
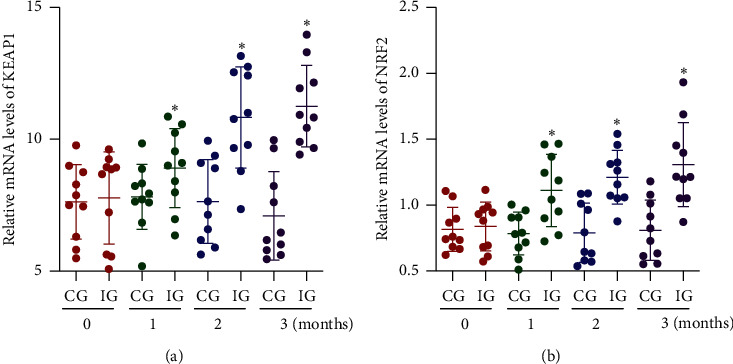
Relative mRNA levels of KEAP1 and NRF2 in the peripheral blood leukocytes (PBLs) of the patients with CHD: (a) KEAP1, Kelch-like ECH-associated protein; (b) NRF2, 1-NF-E2-related factor 2. IG, Antarctic krill oil intervention group; CG, a common-care group. *n* = 10 in each group, and intervention duration was 3 months. The statistical difference was significant if *p* < 0.05.

Before AKO intervention, the statistical difference for relative mRNA levels of KEAP1 (Figure 5(a)) and NRF2 (Figure 5(b)) was insignificant between two groups (*p* > 0.05). After AKO intervention, relative mRNA levels of KEAP1 (Figure 5(a)) and NRF2 (Figure 5(b)) increased in the IG group and higher than those in the CG group (*p* < 0.05). The results suggest that AKO intervention may affect antioxidant capacities by increasing relative mRNA levels of KEAP1 and NRF2 levels in CHD patients.

### 3.6. AKO Increased Relative Protein Levels of KEAP1 and NRF2 in CHD Patients

**Figure 6:**
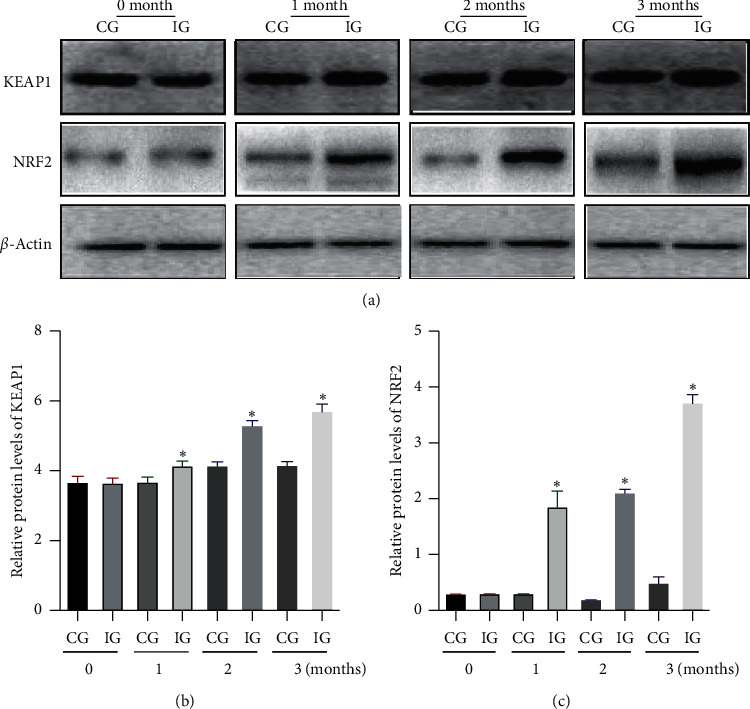
Relative protein levels of KEAP1 and NRF2 in the peripheral blood leukocytes (PBLs) of the patients with CHD: (a) KEAP1, Kelch-like ECH-associated protein; (b) NRF2, 1-NF-E2-related factor 2. IG, Antarctic krill oil intervention group; CG, a common-care group. *n* = 10 in each group, and intervention duration was 3 months. The statistical difference was significant if *p* < 0.05.

Before AKO intervention, the statistical difference for relative protein levels of KEAP1 (Figure 6(a)) and NRF2 (Figure 6(b)) was insignificant between two groups (*p* > 0.05). After AKO intervention, relative protein levels of KEAP1 (Figure 6(a)) and NRF2 (Figure 6(b)) increased in the IG group and higher than those in the CG group (*p* < 0.05). The results suggest that AKO intervention increases antioxidant capacities by increasing relative protein levels of KEAP1 and NRF2 levels in CHD patients.

**Figure 7:**
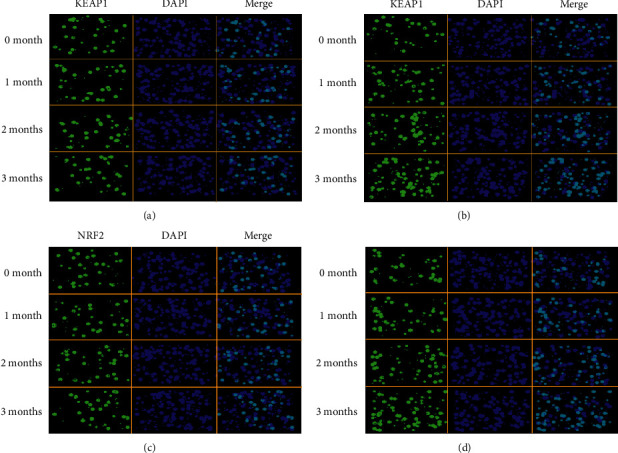
Immunofluorescence staining of peripheral blood leukocytes (PBLs): (a) the expression of KEAP1; (b) the expression of NRF2. The fluorescence localization of KEAP1 and NRF2 was measured with immunofluorescence. Anti-NRF2 and KEAP1 antibodies were used to detect NRF2 and KEAP1 localization (green) using a fluorescence microscope. DAPI staining indicated the locations of the nuclei (blue). *n* = 10 in each group, and intervention duration was 3 months. (a) KEAP1 expression in the CG group. (b) KEAP1 expression in the IG group. (c) NRF2 expression in the CG group. (d) NRF2 expression in the IG group.

Fluorescence staining also showed the similar results as Western blot analysis. The intensity of KEAP1 fluorescence were similar before and after 1, 2, and 3 months of intervention in the CG group (Figure 7(a)). In contrast, the intensity of KEAP1 fluorescence increased and reached highest level after 3 months of AKO intervention in the IG group (Figure 7(b)). Similarly, the intensity of NRF2 fluorescence was similar before and after 1, 2, and 3 months of intervention in the CG group (Figure 7(c)). In contrast, the intensity of NRF2 fluorescence increased and reached highest level after 3 months of AKO intervention in the IG group (Figure 7(d)). The results also suggest that AKO intervention increases protein levels of KEAP1 and NRF2 levels in CHD patients.

### 3.7. AKO Consumption Increased Serum UFA Contents and Reduced Serum SFA Contents

Before AKO intervention, the statistical difference for the serum contents of SFA, MUFA, PUFA, and PUFA-3 was insignificant between the two groups (Table 3, *p* > 0.05). After 3 months of AKO intervention, the serum contents of SFA in the IG group were higher than those in the CG group, whereas the serum contents of MUFA, PUFA, and PUFA-3 had reverse results (Table 3, *p* < 0.05). The results suggest that AKO intervention increases serum UFA contents and reduces serum SFA contents in CHD patients.

### 3.8. ROS Levels Had a Negative Relationship with Serum Levels of EPA or DHA

**Figure 8:**
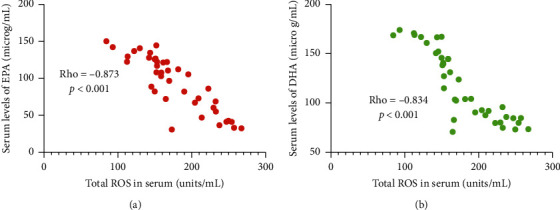
The test of Spearman rank-order correlation coefficient for the relation between the levels of ROS and serum levels of EPA or DHA: (a) EPA; (b) DHA. Rho < −0.5 means a strong negative correlation between two variables.

The test of Spearman rank-order correlation coefficient showed that with the increase in serum SOD, the levels of serum EPA reduced (Figure 8(a), *p* < 0.001). Similarly, the levels of serum DHA reduced (Figure 8(b), *p* < 0.001) with the increase in the total SOD in serum. ROS levels had a negative relationship with serum levels of EPA or DHA, suggesting that EPA and DHA may reduce the total ROS in serum and have antioxidant capacities.

### 3.9. ROS Levels Had a Negative Relation with Relative Protein Levels of KEAP1 or NRF2

**Figure 9:**
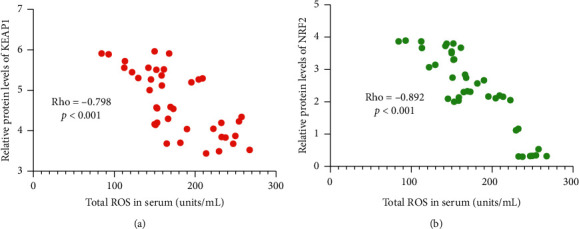
The test of Spearman rank-order correlation coefficient for the relation between the levels of ROS and relative protein levels of KEAP1 and NRF2: (a) KEAP1; (b) NRF2. Rho < −0.5 means a strong negative correlation between the two variables.

The test of Spearman rank-order correlation coefficient showed that with the increase in serum SOD, relative protein levels of KEAP1 reduced (Figure 9(a), *p* < 0.001). Similarly, the levels of NRF2 also reduced (Figure 9(b), *p* < 0.001) with the increase in serum SOD. ROS levels had a negative relation with relative protein levels of KEAP1 and NRF2, suggesting that EPA and DHA may reduce serum ROS by affecting KEAP1 and NRF2 signaling.

## 4. Discussion

The present work showed that AKO contains high levels of EPA and DHA (Table 1). AKO consumption improved lipid contents by reducing serum contents of SFA and increasing serum contents of MUFA and PUFA (Table 3). AKO intervention increased antioxidant capacity in the CHD patients by reducing the serum levels of oxidative biomarkers ROS (Figure 2), 8-OHdG, NO, and MDA (Figure 3), and increasing the levels of antioxidant biomarkers SOD, GSH, and GPx (Figure 3). Meanwhile, AKO treatment also activated antioxidant signaling KEAP1 and NRF2 in PBL (Figures 5–7). Serum levels of EPA and DHA had a strong negative relationship with the level of ROS (Figure 8), whereas ROS had a strong negative relation with the levels of KEAP1-NRF2 (Figure 9). Thus, all these findings imply that AKO may improve antioxidant capacities in CHD patients by activating KEAP1 and NRF2 signaling via EPA and DHA.

AKO addition in the diet reduced serum contents of SFA and increased serum contents of MUFA and PUFA in CHD patients. The changes may be caused by the high levels of UFA and lower levels of SFA in AKO. AKO increased the antioxidant capacities in the CHD patients via EPA and DHA, which were consistent with that EPA and DHA had high levels of antioxidant activities [36, 37]. EPA has been found to show the greatest antioxidant activity by reducing 70% MDA level, followed by DHA, 2 of which have higher antioxidant activities than other omega-3 FA [38]. EPA and DHA should play important roles in the antioxidant activity of AKO.

KEAP1 and NRF2 are associated with antioxidant function [39], and activation of KEAP1/NRF2 signaling results in the increase of antioxidant activities [40]. However, the effects of EPA and DHA on KEAP1/NRF2 signaling remain widely unknown. The correction test indicated that ROS levels had negative relation with serum levels of EPA and DHA (Figure 8), and the levels of KEAP1 and NRF2 (Figure 9) in the CHD patients. Thus, EAP and DHA levels may have a strong relationship with the KEAP1 and NRF2 levels. Further work is needed to be done to confirm the conclusion.

## 5. Limitations of This Study

This study was a small sample and single-center study, and the research results may have a certain degree of bias. In order to control the bias, all the subjects in this study were from the same place and recruited in the same department of our hospital. The indicators after the patient were not studied for more than 3 months, so the long-term effect remains unclear. Total antioxidant capacity (TAC) and catalase are important other biomarkers [41] and were not studied in the present work. EPA and DHA have been found to have strong anti-inflammatory effects [42], but the serum levels of inflammatory factors were not measured either. Much work is highly demanded to address these important issues.

## Figures and Tables

**Table 1:** Fatty acid composition of Antarctic krill oil extracted with different solvents.

Fatty acids	Content percent	Types
C12 : 0	0.22 ± 0.05	SFA
C14 : 0	10.25 ± 0.23	SFA
C14 : 1	0.21 ± 0 01	MUFA
C15 : 0	0.45 ± 0.03	SFA
C16 : 0	21.99 ± 0.11	SFA
C16 : 1	4.79 ± 0.05	MUFA
C17 : 0	0.14 ± 0.02	SFA
C17 : 1	0.20 ± 0.02	MUFA
C18 : 0	1.19 ± 0.15	SFA
C18 : 1n9t	10.83 ± 0.17	MUFA
C18 : 1n9c	7.67 ± 0.12	MUFA
C18 : 2n6t	0.30 ± 0.01	PUFA
C18 : 2n6c	2.01 ± 0.11	PUFA
C18 : 3n6	0.32 ± 0.02	PUFA
C18 : 3n3	2.30 ± 0.03	PUFA
C20 : 1	6.27 ± 0.12	MUFA
C21 : 0	0.18 ± 0.02	SFA
C20 : 4n6	0.37 ± 0.01	PUFA
C20 : 3n3	0.18 ± 0.02	PUFA
C22 : 1	0.69 ± 0.05	MUFA
C20 : 5n3	17.40 ± 0.17	PUFA
C22 : 6n3	11.26 ± 1.04	PUFA
SFA	34.42 ± 0.08	—
MUFA	30.95 ± 0.23	—
PUFA	34.63 ± 0.21bc	—
PUFA *n*−3	31.63 ± 0.19b	—

Note: SFA, saturated fatty acid; MUFA, monounsaturated fatty acid; PUFA, polyunsaturated fatty acid.

**Table 2:** Baseline characteristics between the two groups.

Parameters	IG	CG	t/*χ*^2^/f-ratio	*p* values
Age (age)	60.30 ± 8.40	58.56 ± 12.23	0.625^a^	0.525
Male, *n* (%)	26 (52.00)	24 (48.00)	1.920^b^	0.383
Smoking, *n* (%)	25 (50.00)	22 (44.00)	0.361^b^	0.548

Education level, *n* (%)				
Junior high school and below	30 (60.00)	35 (70.00)	1.099^b^	0.295
High school and above	20 (40.00)	15 (30.00)	—	—

BMI (kg/m^2^)	23.95 ± 3.16	23.64 ± 4.06	0.091^a^	0.913

CSA, cases (%)				
Grade 0	4 (8)	3 (6)	0^c^	1
Grade I	4 (8)	6 (12)		
Grade II	28 (56)	25 (50)		
Grade III	12 (24)	13 (26)		
Grade IV	2 (4)	3 (6)		

Drug history, cases (%)				
Antilipidemic	6 (12)	5 (10)	0^c^	1
Hypoglycemic	2 (4)	3 (6)		
Nitrates	10 (20)	11 (22)		
Beta-blocker	24 (48)	22 (44)		
Calcium antagonists	3 (6)	5 (10)		
ACE-inhibitors	4 (8)	3 (6)		
Diuretics	1 (2)	1 (2)		

Note: CAS, coronary artery spasm, refers to a sudden, intense vasoconstriction of an epicardial coronary artery which results in vessel occlusion or near occlusion. CAS was measured by 18F-fluorodeoxyglucose (18F-FDG). a stands for *t* values, b stands for *χ*^2^ values, and c stands for f-ratio. *n* = 50 in each group, and the statistical difference was insignificant if *p* > 0.05.

**Table 3:** The comparison of fatty acids between the IG and CG groups.

Fatty acids (% of total fatty acids)	IG	CG	*p* values
Before intervention			
SFA	33.2 (30.2, 35.6)	32.7 (29.4, 35.1)	0.651
MUFA	24.5 (20.3, 26.7)	25.3 (21.4, 27.8)	0.529
PUFA	42.9 (37.1, 48.5)	42.0 (37.8, 49.0)	0.714
PUFA *n*−3	7.8 (5.3, 9.6)	8.2 (5.6, 10.4)	0.806
After intervention			
SFA	29.3 (27.4, 33.1)	35.6 (30.2, 36.7)	0.007
MUFA	27.1 (22.5, 29.2)	25.0 (20.9, 27.3)	0.015
PUFA	43.6 (38.3, 49.7)	39.4 (36.1, 48.5)	0.038
PUFA *n*−3	8.1 (5.5, 9.8)	7.2 (5.2, 9.6)	0.032

Note: the median in the brackets was given from 25^th^ to 75^th^ percentile. MUFA, monounsaturated fatty acid; PUFA, polyunsaturated fatty acid; SFA, saturated fatty acid.

## Data Availability

The datasets generated and analyzed in this study are not publicly available because patient information is contained in the data, but all data are available from the correspondent author on reasonable request.
